# A deep learning phenome wide association study of the electrocardiogram

**DOI:** 10.1093/ehjdh/ztaf047

**Published:** 2025-05-08

**Authors:** John Weston Hughes, John Theurer, Milos Vukadinovic, Albert J Rogers, Sulaiman Somani, Guson Kang, Zaniar Ghazizadeh, Jack W O’Sullivan, Sneha S Jain, Bruna Gomes, Michael Salerno, Euan Ashley, James Y Zou, Marco V Perez, David Ouyang

**Affiliations:** Department of Computer Science, Stanford University, 353 Jane Stanford Way, Stanford, CA 94305, USA; Department of Cardiology, Cedars-Sinai Medical Center, Smidt Heart Institute, Los Angeles, CA, USA; Department of Cardiology, Cedars-Sinai Medical Center, Smidt Heart Institute, Los Angeles, CA, USA; Department of Bioengineering, University of California, Los Angeles, Los Angeles, CA, USA; Department of Medicine, Stanford University, Stanford, CA, USA; Department of Medicine, Stanford University, Stanford, CA, USA; Department of Medicine, Stanford University, Stanford, CA, USA; Department of Medicine, Stanford University, Stanford, CA, USA; Department of Medicine, Stanford University, Stanford, CA, USA; Department of Medicine, Stanford University, Stanford, CA, USA; Department of Medicine, Stanford University, Stanford, CA, USA; Department of Medicine, Stanford University, Stanford, CA, USA; Department of Medicine, Stanford University, Stanford, CA, USA; Department of Biomedical Data Science, Stanford University, Stanford, CA, USA; Department of Medicine, Stanford University, Stanford, CA, USA; Department of Cardiology, Cedars-Sinai Medical Center, Smidt Heart Institute, Los Angeles, CA, USA

**Keywords:** Electrocardiogram, Phewas, Disease screening, Artificial intelligence

## Abstract

**Aims:**

Deep learning methods have shown impressive performance in detecting a range of diseases from electrocardiogram (ECG) waveforms, but the breadth of diseases that can be detected with high accuracy remains unknown, and in many cases the changes to the ECG allowing these classifications are also opaque. In this study, we aim to determine the full set of cardiac and non-cardiac conditions detectable from the ECG and to understand which ECG features contribute to the disease classification.

**Methods and results:**

Using large datasets of ECGs and connected electronic health records from two separate medical centres, we independently trained PheWASNet, a multi-task deep learning model, to detect 1243 different disease phenotypes from the raw ECG waveform. We confirmed that the ECG can be used to detect chronic kidney disease (AUC = 0.80), cirrhosis (AUC = 0.80), and sepsis (AUC = 0.84), as well as a range of cardiac diseases, and also found new detectable conditions, including respiratory failure (AUC = 0.86), neutropenia (AUC = 0.83), and menstrual disorders (AUC = 0.84). We found that of the 37 non-cardiac strongly detectable conditions, 35 were detectable by the model output for just four diseases, suggesting that they have similar effects on the ECG. We found that high performance in some conditions including neutropenia, respiratory failure, and sepsis can be explained by linear models based on conventional measurements taken from the ECG.

**Conclusion:**

Our study uncovers a range of diseases detectable in the ECG, including many previously unknown phenotypes, and makes progress towards understanding ECG features that allow this detection.

## Graphical Abstract

**Graphical Abstract:**
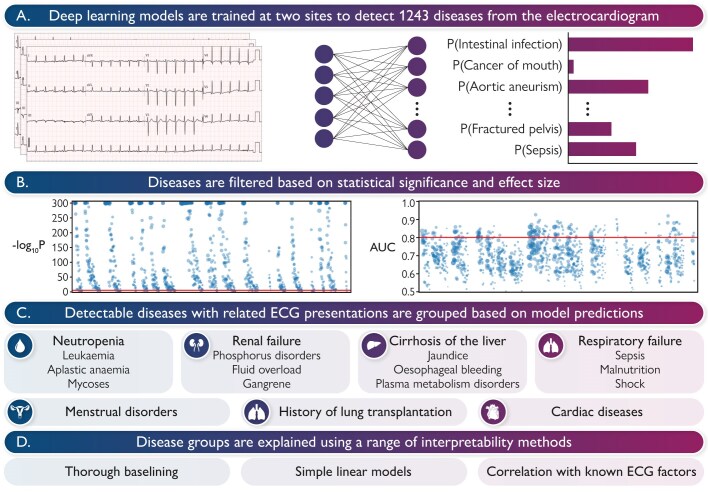

## Introduction

The electrocardiogram (ECG) is an inexpensive and ubiquitous cardiovascular diagnostic tool. Recently, deep learning methods have demonstrated impressive performance in evaluating cardiovascular health from the ECG, from outperforming cardiologists at basic overreading^1^ to detecting subtle phenotypes like left ventricular systolic dysfunction,^2,3^ valvular disease,^4^ and atrial fibrillation in sinus rhythm^5^ to predicting risk of future cardiovascular and all-cause mortality.^6–8^ These findings, while impressive and useful, are in a sense straight-forward: a powerful new analytic framework has unlocked new cardiac information in a cardiac diagnostic tool. Deep learning models using the ECG have also been shown to detect ‘hidden phenotypes’ and diagnoses not traditionally inferred by Cardiologists from the ECG or directly related to cardiac health, such as age and sex,^9^ chronic kidney disease,^10^ cirrhosis of the liver,^11^ sepsis,^12^ hypokalaemia and hyperkalaemia,^13,14^ anaemia,^15^ hyperthyroidism,^16^ and COVID-19.^17^

The presence of ECG-assessable hidden phenotypes raises several interesting questions. What is the full range of these hidden phenotypes? What information in the ECG reveals these phenotypes? Is that information common or unique to each and is it human understandable? Answering these questions could unlock a set of potentially clinically useful screening tools for non-cardiac disease and broaden our understanding of the ECG and the relationship between the heart and other organ systems. Such tools could be used to identify patients at high risk for disease, whether they receive an ECG in the clinic or via a smartwatch or other mobile health device.

Similar questions led geneticists to develop the phenome-wide association study, or PheWAS, which tests the association between a single nucleotide polymorphism and a wide range of phenotypes.^18,19^ This idea has recently been extended to other quantitative measurements like retinal optical coherence tomography images, unearthing relationships between retinal thickness and cardiac, pulmonary, and neurological conditions.^20^ In our previous work, we took this approach a step further and implemented a deep learning model to detect fourteen different abnormal laboratory values from the echocardiogram, discovering new associations with anaemia and increased blood urea nitrogen.^21^ These tools allow for discovery of interesting associations, but as with all deep learning methods, interpretability can pose a major challenge. To mitigate this, we previously developed simple linear models based on ECG measurements to achieve similar results to deep learning.^22^

In this study, we set out to investigate three questions. First, which diseases are detectable from the ECG using deep learning? We train PheWASNet, a multi-task deep learning model, to detect 1243 different disease phenotypes from the ECG waveform, as well as a host of baselines to allow us to understand model performance in context. Second, which of these diseases have similar effects on the ECG? We evaluate how well detectors for one disease detect another to understand whether the salient ECG features used for multiple diseases are the same. Third, which of these effects can be simply explained? We employ a toolbox of simple linear models, odds ratios, and demographic correlations to understand how the ECG allows detection of different diseases. (*Graphical abstract*) shows a visual representation of the questions tackled in this study.

## Methods

### Study populations and data sources

We trained and evaluated PheWASNet models independently on ECGs from two medical systems, Stanford Healthcare and Cedars Sinai Medical Centre, to explore generalisable ECG-disease associations. At Stanford we used all ECGs recorded during clinical care between August 2005 and May 2018, associated with electronic health record data from the same period. ECGs were recorded using Philips ECG machines and extracted using the Philips TraceMaster software, and were all 10 s long at 500 Hz, downsampled to 250 Hz. We applied band pass and wandering baseline filters to the signals and normalized on a per-lead basis. Diagnosis codes from the ICD-9 and ICD-10 ontologies were extracted using STARR-OMOP,^23^ a common data model copy of Stanford’s electronic health record system. Similarly at Cedars Sinai, ICD-9 and ICD-10 codes were extracted directly from the electronic health record, while ECGs were recorded using General Electric ECG machines and extracted using the MUSE Cardiology Information System. At both sites, patients were randomly split into training, tuning, and test splits by patient in a 5:1:4 ratio such that no patient was in multiple splits. At Stanford, we additionally acquired a list of 555 measurements automatically taken by the Philips TraceMaster software from the ECG, as well as the clinician text interpretations which were parsed into tabular labels using string matching.

Diagnosis codes and ECGs were associated if they occurred within 30 days of each other. If a patient received multiple ECGs within 30 days of a single diagnosis code, the diagnosis applied to all such ECGs, and if multiple diagnosis codes occurred within 30 days of an ECG, all those diagnosis codes applied to that ECG. The ICD-9 and ICD-10 codes were translated into phecodes,^19,24^ a list of 1868 hierarchical categories of disease diagnosis code, which cover all ICD codes and are designed specifically for PheWAS studies. Of those 1,868, we included in our labels those which had at least 100 total positive ECGs and 10 within each of the training, tuning, and test sets. This set of labels was used to train the PheWASNet models.

The Stanford University Institutional Review Board approved this study under protocol 41045 and it complied with all relevant ethical regulations; the review board waived the requirement for informed consent owing to the retrospective nature of the data and project.

### Model design

PheWASNet models were trained to intake 12-lead ECGs and detect all 1243 phenotypes in a multi-task learning setup, where a model backbone was shared across all tasks and independent final layers were trained to detect each phenotype. A multilabel logistic loss was used, minimising the sum of binary cross-entropies between labels and model outputs across all phenotypes. The PheWASNet models consisted of a 1d EfficientNet architecture similar to that described in our previous work,^7,10,25^ consisting of 4 407 507 parameters of which 1 592 283 were in the final layer, contributing 1281 parameters to each phenotype task. The full model architecture is described in supplementary material online, *Figure S3*. All models were trained on the training set and all hyperparameters and design decisions were tuned based on results on the tuning set. Results were only evaluated once on the test set to avoid overfitting. We found that overall results were largely invariant to choice of hyperparameters, and particularly that loss-weighting to correct for class imbalance did not have a significant impact. All PheWASNet models were trained on 12-lead ECG waveforms unless otherwise specified. Single lead comparison models were trained using the same architecture and hyperparameters, replacing 12 channel convolutions with single channel and passing only lead I of the ECG as input. Models were developed using Python 3.9 and PyTorch 1.11 on single Nvidia Titan Xp GPUs using Stanford’s Sherlock computing cluster.

### Statistical analysis

We primarily compared models based on *P*-values and AUROC. The AUROC is a standard metric for determining the performance of a discriminative model across multiple sensitivity and specificity cutoffs. To identify diseases for further analysis, we used a multi-step hypothesis testing framework. We first identified the set of phenotypes which the model could detect with statistically significant performance. Using a two-tailed non-parametric Mann–Whitney *U* test,^26^ we evaluated the null hypothesis that model predictions for positive and negative ECGs were drawn from the same distribution against the alternate hypothesis that the sum of scores for one group was greater than the other. We repeated the test for each condition. We computed *P*-values using a Bonferroni correction to achieve a false discovery rate of below 1% (0.01/1243 ≈ 8 × 10^−6^). We then additionally filtered phenotypes which had fewer than 500 corresponding ECGs. We performed this filtering first at Stanford, generating a list of potentially relevant diseases, and then to that list applied the same filtering at Cedars-Sinai. To find a shortlist of very detectable diseases, we additionally filtered to only consider phenotypes for which PheWASNet first achieved an AUC of 0.8 at Stanford and second achieved an AUC of 0.75 at Cedars Sinai. A lower cutoff was used at Cedars-Sinai to account for close cases where the AUC was above 0.8 at Stanford but just below 0.8 at Cedars-Sinai due to statistical noise. We performed more targeted analyses on the diseases remaining after this filtering. While appropriate AUROC cutoffs can vary depending on downstream clinical application, there is reasonable consensus that a score of above 0.8 should be considered good or excellent.^27^ We additionally computed the cutoff in model predictions yielding the specificity closest to 90%, and computed specificity, sensitivity, positive predictive value, F1 score, and confusion matrix statistics at that cutoff in the Stanford dataset for the PheWASNet model. We also include areas under the precision-recall curve.

### Efforts in interpretability

The performance of a single model class across a wide range of classes can be difficult to understand in isolation. To better understand what features of the input are useful in detecting different phenotypes and what pathways the model might be using, we designed a number of baselines to evaluate how difficult different diagnoses are to detect. We first compared the PheWASNet model to a number of univariate predictors: age; the previously published SEER score,^6^ a deep learning predictor of the long-term risk of cardiovascular mortality; and the three ECG measurements with the highest average performance across phenotypes, heart rate, corrected QT interval, and the area under the T-wave in aVR. Next, we compared a linear model based on age, race, ethnicity, gender, and BMI, all of which have been shown to be associated with various ECG features.^9,28,29^ Finally, we trained ridge regression and random forest models based on 555 extracted ECG measurements. Baseline models were trained using Python 3.9, XGBoost 1.7, and Scikit-Learn 1.2. We tuned the regularisation hyperparameter for linear models and the depth and number of trees for the XGBoost models, using the binary logistic loss.

To understand how different conditions within the dataset are related, we examined how well the detector for one phecode could detect other phecodes. Among the 37 non-cardiovascular phenotypes for which the PheWASNet model achieved an AUC of above 0.8 in the Stanford test set and 0.75 in the Cedars Sinai test set, we considered which phenotypes could be well-detected by the model prediction for another phenotype. We considered two phenotypes to be ‘co-detectable’ if the detector for each condition can detect the other condition with an AUC of no more than 0.05 lower than the other condition’s own detector (see Supplementary material online, *Figure S1*). For example, the PheWASNet model detected respiratory failure with an AUC of 0.86 and sepsis with an AUC of 0.84. If you use the model’s output for respiratory failure to detect sepsis, or the model’s output for sepsis to detect respiratory failure, you also achieve an AUC of 0.84. Thus, we refer to the two as co-detectable. Two diseases being co-detectable might suggest that they have similar effects on the ECG, possibly because presence of the diseases is correlated and possibly because they affect the heart in similar ways. Conversely, co-detectability might imply that it is difficult to distinguish between two diseases using the ECG.

Next, we set out to understand how each group of co-detectable diseases could be identified in the ECG. We first considered the baselines discussed above to find phenotypes or groups of phenotypes which were well-explained by those baselines. Next, we explored training simpler linear models for a subset of conditions following our previous work.^22^ We applied lasso regularisation and hand-examination of variables to move from models on 555 ECG-based variables to between two and seven, following similar training protocols to the large linear models. Once the shortlist of variables was selected, another model was trained without regularisation on the same dataset to achieve optimal performance. Third, we computed the odds ratios of both a condition occurring and an ECG being in the top 5% of risk for that condition given various ECG interpretation statements, and reviewed the top odds ratios.

## Results

### Study populations

A summary of demographics and population sizes is shown in Supplementary material online, *Table S1*. The Stanford cohort consisted of 954 817 ECGs from 318 316 patients, split into a train set of 382 729 ECGs (127 193 patients), a tuning set of 95 654 (32 005), and a test set of 476 434 (159 118). The average patient was 61 with a standard deviation of 17, and 56.7% of patients were non-Hispanic White, 10.3% Asian, 5.4% Black, and 2.2% Hispanic White. ECGs had on average 4.1 positive corresponding phenotypes (median 2) while phenotypes had on average 1593.4 corresponding ECGs (median 441). The Cedars-Sinai cohort consisted of 1 346 791 ECGs (542 858 patients), split into 538 091 (213 298) training examples, 134 455 (88 143) tuning, and 674 245 (241 417) test. That cohort had 16.4% Black and 10.2% Hispanic White patients. The were an average of 9.8 positive corresponding phenotypes (median 6) for each ECG and Cedars-Sinai, while each phenotype had on average 7046.2 corresponding ECGs (median 956). Of the 1868 total phenotypes, 1243 occurred in more than 100 ECGs at each site and were used in the training labels. Overlap between conditions in the Stanford cohort is shown in Supplementary material online, *Figure S2*.

### The PheWASNet model detects a wide range of disease from the ECG

Of the 1243 diseases considered, the PheWASNet model detected 1110 with statistically significant performance. 585 of those had at least 500 corresponding ECGs at Stanford. After applying the same filters at Cedars-Sinai, 566 diseases remained. We focus our analyses on these 566 diseases, and include all ECGs, including ECGs not associated with any phecode, in analyses.

**Figure 1:**
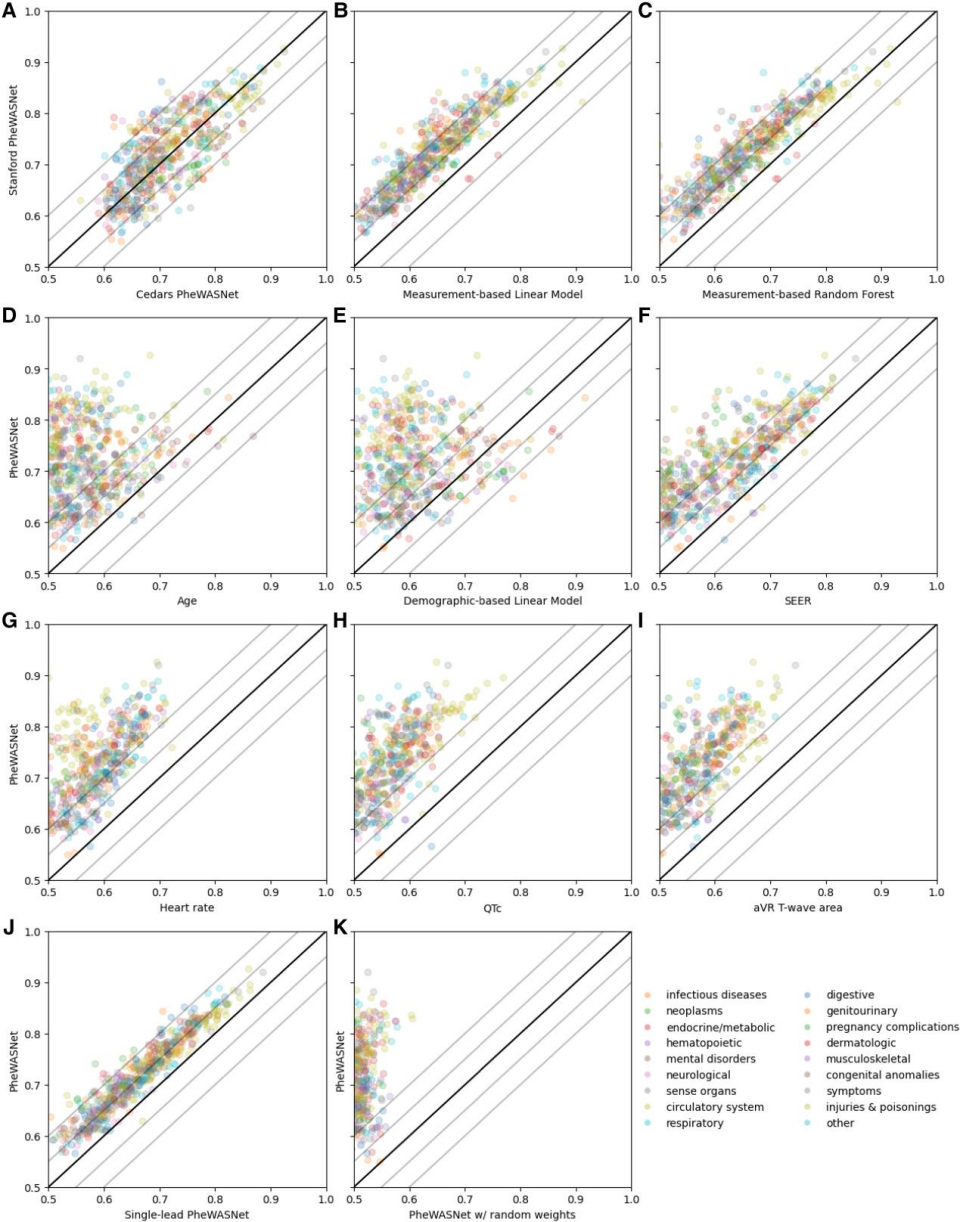
Comparison of PheWASNet to baselines. Colours represent categories. All comparisons are performed at Stanford unless noted. (*A*) Stanford vs. Cedars-Sinai performance. (*B–C*) Comparison to models based on 555 measurements from the ECG. (*D*) Comparison to age as a univariate predictor. (*E*) Comparison to a linear model using age, sex, race/ethnicity, and BMI. (*F*) Comparison to SEER as a univariate predictor. (*G–I*) Comparison to three ECG measurements as univariate predictors. (*J*) Comparison between 12 and 1-lead PheWASNet models. (*K*) Comparison to a PheWASNet model with randomly initialized weights.

**Figure 2:**
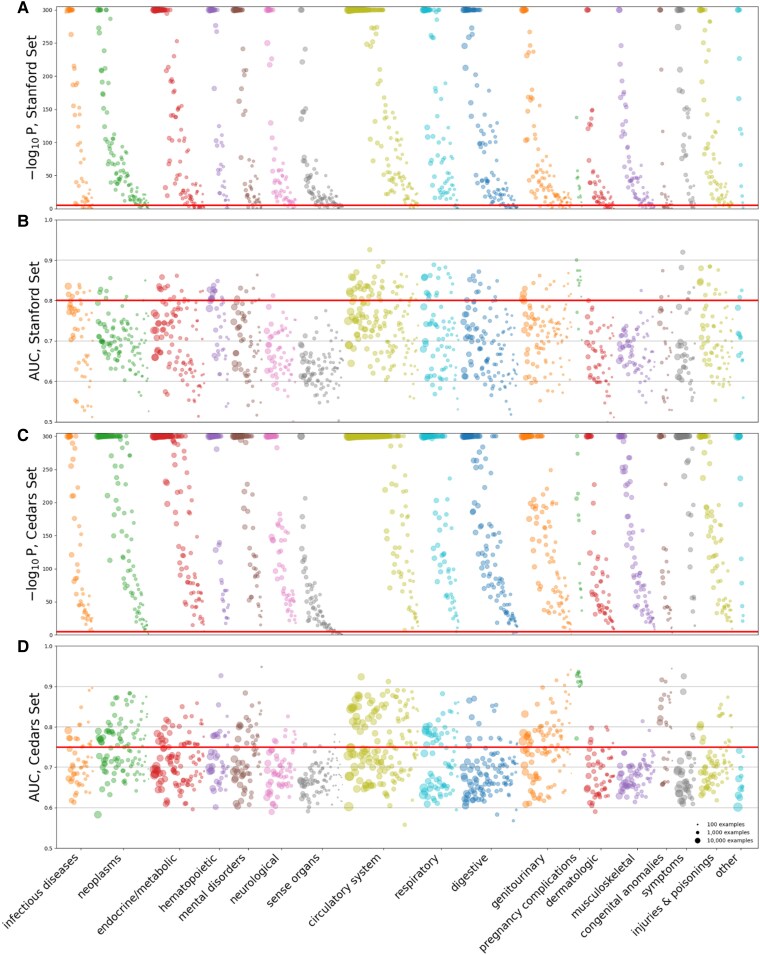
(*A*) Visualisation of statistical significance of model performance across 1243 phenotypes in the Stanford cohort. Colours represent categories and areas of dots represent the number of examples in the test set. The bold line marks the cutoff for a false discovery rate of 1%. (*B*) AUCs in the Stanford cohort. The red line marks the cutoff for an effect size cutoff of 0.80. (*C–D*) The same for the Cedars-Sinai cohort.

Of the 566 phenotypes considered, we were able to detect 78 with an AUC above both 0.8 at Stanford Medical Centre and 0.75 at Cedars-Sinai Medical Centre. *Table 1* shows a list of these phenotypes with their AUC and count at each site and their category. Supplementary material online, *Figure S1* visually shows the AUCs of all 566 phenotypes ordered by organ system and count for both sites, and Supplementary material online, *Table S3* summarizes all results. Supplementary material online, *Figure S4* shows ROC curves for eight selected conditions along with sensitivities achieved at key specificity thresholds. Notably, while the cardiovascular system was the largest category considered and many of the most-easily detectable phenotypes were cardiovascular, other categories also had highly-detectable phenotypes. *Figure 1A* shows the relationship between Stanford and Cedars-Sinai ECGs, which were for the most part similar, with 67.0% of phecode AUCs within 5% between Stanford and Cedars and 94.0% within 10%. Among those phenotypes where the Stanford and Cedars Sinai models differed by more than 10%, in 1.6% of cases the Cedars-Sinai model performed better, and in 4.4% of cases the Stanford model performed better. Among the 78 considered high-performing phenotypes, 100% of AUCs were within a 10% margin at Stanford and Cedars-Sinai. Supplementary material online, *Table S3* lists all 566 phenotypes and their performance at each site. *Figure 2* shows *P*-values and AUCs across the spectrum of disease.

**Table 1:** Model performance at Stanford and cedars-Sinai on all phenotypes with a Stanford AUC above 0.8 and a Cedars-Sinai AUC above 0.75 and more than 500 examples at each site

			Stanford		Cedars-Sinai	
Phecode	Phenotype	Category	Test AUC	Test count	Test AUC	Test count
038	Septicaemia	Infectious diseases	0.84	17 133	0.79	26 357
038.1	Gram negative septicaemia	Infectious diseases	0.82	711	0.77	1166
117	Mycoses	Infectious diseases	0.81	1666	0.77	1792
204.11	Lymphoid leukaemia, acute	Neoplasms	0.86	866	0.85	878
204.2	Myeloid leukaemia	Neoplasms	0.82	2285	0.79	2618
204.21	Myeloid leukaemia, acute	Neoplasms	0.83	2124	0.83	2024
260	Protein-calorie malnutrition	Endocrine/metabolic	0.84	5206	0.76	12 216
260.2	severe protein-calorie Malnutrition	Endocrine/metabolic	0.84	1463	0.80	2097
270.38	Other specified disorders of Plasma protein metabolism	Endocrine/metabolic	0.82	562	0.76	502
275.53	Disorders of phosphorus Metabolism	Endocrine/metabolic	0.81	923	0.76	1551
276.11	Hyperosmolality and/or hypernatremia	Endocrine/metabolic	0.86	3727	0.80	3147
276.6	Fluid overload	Endocrine/metabolic	0.83	2231	0.81	4714
277.4	Disorders of bilirubin excretion	Endocrine/metabolic	0.80	1258	0.76	1965
284	Aplastic anaemia	Hematopoietic	0.82	2196	0.78	5357
284.1	Pancytopenia	Hematopoietic	0.82	2103	0.78	5177
286.7	Other and unspecified coagulation defects	Hematopoietic	0.84	2904	0.78	2477
288.11	Neutropenia	Hematopoietic	0.83	4082	0.78	3641
290.2	Delirium due to conditions Classified elsewhere	Mental disorders	0.83	2063	0.78	1062
317.11	Alcoholic liver damage	Mental disorders	0.82	765	0.88	2820

			Stanford		Cedars-Sinai	
Phecode	Phenotype	Category	Test AUC	Test count	Test AUC	Test count
394	Rheumatic disease of the heart valves	Circulatory system	0.84	2500	0.82	12 706
394.2	Mitral valve disease	Circulatory system	0.83	1121	0.83	7519
394.7	Disease of tricuspid valve	Circulatory system	0.85	1124	0.86	4214
395.2	Non-rheumatic aortic valve Disorders	Circulatory system	0.83	8296	0.83	31 621
395.6	Heart valve replaced	Circulatory system	0.83	1161	0.85	23 782
411.2	Myocardial infarction	Circulatory system	0.81	9809	0.80	33 618
411.8	Other chronic ischaemic heart Disease, unspecified	Circulatory system	0.83	2112	0.86	11 835
415.2	Chronic pulmonary heart disease	Circulatory system	0.84	3672	0.81	10 075
425	Cardiomyopathy	Circulatory system	0.83	6398	0.87	23 609
425.1	Primary/intrinsic Cardiomyopathies	Circulatory system	0.84	5434	0.88	22 698
426.2	AV block	Circulatory system	0.84	2923	0.85	5823
426.24	AV block, complete	Circulatory system	0.88	1545	0.89	3209
426.3	Bundle branch block	Circulatory system	0.86	1845	0.88	5143
426.31	Right bundle branch block	Circulatory system	0.82	534	0.88	1857
426.32	Left bundle branch block	Circulatory system	0.90	1118	0.91	2913
427.12	Paroxysmal ventricular Tachycardia	Circulatory system	0.83	4688	0.83	11 031
427.2	Atrial fibrillation and flutter	Circulatory system	0.82	32 572	0.85	73 782
427.21	Atrial fibrillation	Circulatory system	0.82	28 383	0.85	68 895
427.22	Atrial flutter	Circulatory system	0.83	5419	0.87	10 632
427.4	Cardiac arrest and ventricular Fibrillation	Circulatory system	0.82	1921	0.84	9816
427.42	Cardiac arrest	Circulatory system	0.81	1527	0.84	9248
427.8	Sinoatrial node dysfunction (Bradycardia)	Circulatory system	0.80	1375	0.85	4641
428	CHF; Non-hypertensive	Circulatory system	0.86	21 132	0.84	84 689
428.1	CHF NOS	Circulatory system	0.86	12 422	0.85	51 105
428.2	Heart failure NOS	Circulatory system	0.84	1886	0.85	8484
428.3	Heart failure with reduced EF [Systolic or combined heart failure]	Circulatory system	0.87	6831	0.87	33 857
428.4	Heart failure with preserved EF [Diastolic heart failure]	Circulatory system	0.81	2420	0.79	21 183
429.1	Heart transplant/surgery	Circulatory system	0.93	2210	0.92	19 400
442.1	Aortic aneurysm	Circulatory system	0.80	5337	0.76	8090
480.1	Bacterial pneumonia	Respiratory	0.81	2485	0.76	2061

			Stanford		Cedars-Sinai	
Phecode	Phenotype	Category	Test AUC	Test count	Test AUC	Test count
501	Pneumonitis due to inhalation of food or vomitus	Respiratory	0.80	2565	0.79	3147
503	Pulmonary congestion and hypostasis	Respiratory	0.81	1163	0.80	1752
505	Other pulmonary inflamation or oedema	Respiratory	0.82	1070	0.82	2311
506	Empyema and pneumothorax	Respiratory	0.82	2885	0.77	2299
507	Pleurisy; pleural effusion	Respiratory	0.82	5880	0.79	26 836
509	Respiratory failure, insufficiency, Arrest	Respiratory	0.86	15 666	0.79	29 050
509.1	Respiratory failure	Respiratory	0.86	13 104	0.80	21 420
509.2	Respiratory insufficiency	Respiratory	0.86	944	0.78	8064
509.3	Pulmonary insufficiency or respiratory failure following Trauma and surgery	Respiratory	0.86	2076	0.83	1944
510.2	Lung transplant	Respiratory	0.89	1409	0.88	1972
519.2	Respiratory complications	Respiratory	0.88	883	0.84	540
530.2	Oesophageal bleeding (varices/hemorrhage)	Digestive	0.83	617	0.85	984
571	Chronic liver disease and cirrhosis	Digestive	0.80	4839	0.77	10 465
571.8	Liver abscess and sequelae of chronic liver disease	Digestive	0.85	2999	0.87	4914
572	Ascites (non-malignant)	Digestive	0.85	1648	0.87	7343
573.2	Liver replaced by transplant	Digestive	0.84	1100	0.81	3227
573.4	Acute and subacute necrosis of liver	Digestive	0.87	1151	0.85	1093
573.5	Jaundice (not of newborn)	Digestive	0.82	1165	0.77	2987
585	Renal failure	Genitourinary	0.80	32 375	0.76	81 337
585.32	End stage renal disease	Genitourinary	0.83	4036	0.83	24 825
626	Disorders of menstruation and other abnormal bleeding from Female genital tract	Genitourinary	0.84	767	0.86	2063
747.12	Valvular heart disease/heart Chambers	Congenital anomalies	0.81	841	0.87	2417
791	Gangrene	Symptoms	0.82	557	0.78	1369
797	Shock	Symptoms	0.88	3381	0.89	8921
797.1	Cardiogenic shock	Symptoms	0.92	2111	0.93	6341
860	Bone marrow or stem cell Transplant	Neoplasms	0.82	1099	0.79	2241
964	Poisoning by agents primarily affecting blood constituents	injuries and poisonings	0.88	732	0.83	503

			Stanford		Cedars-Sinai	
Phecode	Phenotype	Category	Test AUC	Test count	Test AUC	Test count
994	Sepsis and SIRS	Injuries and poisonings	0.84	11 493	0.80	23 763
994.2	Sepsis	Injuries and poisonings	0.85	10 485	0.81	22 400

Of those 78 phenotypes 29 fell within the cardiovascular category. Several cardiac conduction disorders ranked highly, encompassing various manifestations of bundle branch and atrioventricular (AV) blocks with AUCs between 0.84 and 0.9 at Stanford and 0.85 and 0.91 at Cedars-Sinai. Heart failure conditions also were easily detectable, with AUCs between 0.81 and 0.87 at Stanford and 0.79 and 0.87 at Cedars-Sinai. Heart failure with preserved ejection fraction was the most difficult manifestation of heart failure to detect, while reduced ejection fraction was the easiest (although several percent lower than when defining the same condition based on labels derived from echocardiography among patients who received echocardiograms in the same cohort.^22^) The highest performer was heart transplant/surgery, with AUCs of 0.93 at Stanford and 0.92 at Cedars-Sinai. Valvular diseases were detected with AUCs between 0.82 and 0.86 at both sites, consistent with previous results.^4^ Intrinsic cardiomyopathy was detected with an AUC of 0.84 at Stanford and 0.88 at Cedars-Sinai, while chronic pulmonary heart disease achieved AUCs of 0.84 and 0.81, respectively. Dysrhythmias ranging from atrial fibrillation and flutter to cardiac arrest and ventricular fibrillation achieved relatively modest AUCs between 0.8 and 0.83 at Stanford and 0.83 and 0.87 at Cedar-Sinai, likely due to either their transient nature or imperfect coding. Similarly, myocardial infarction and aortic aneurysm achieved modest results. Notably, Additionally, two conditions not within the cardiovascular category are directly associated with the cardiovascular system: cardiogenic shock and a valvular disease phenotype within the congenital anomalies category. Cardiogenic shock achieved some of the highest AUCs of 0.92 and 0.93.

The next most common category was respiratory with 12 phenotypes. Lung transplant was the easiest respiratory phenotype, achieving AUCs of 0.89 and 0.88 at Stanford and Cedars-Sinai. The non-specific respiratory complications also did well, with AUCs of 0.88 and 0.84. Four different manifestations of respiratory failure all achieved AUCs of 0.86 at Stanford and between 0.78 and 0.83 at Cedars-Sinai. Pleural effusion, pneumothorax, pulmonary oedema, congestion, pneumonitis due to inhalation of food or vomitus, and bacterial pneumonia all achieved modest performance of between 0.8 and 0.82 at Stanford and 0.76 and 0.82 at Cedars-Sinai. Notably there was major overlap between many of the respiratory phenotypes, with all non-respiratory failure/insufficiency/arrest phenotypes: between 26% (for lung transplant) and 59% for respiratory complications also having a diagnosis of respiratory failure/insufficiency/arrest.

Other phenotypes previously known to be associated with the ECG were present. Liver disease phenotypes were common: necrosis of the liver achieved AUCs of 0.87 and 0.85 at Stanford and Cedars-Sinai, while liver transplant achieved AUCs of 0.84 and 0.81. Ascites, oesophageal bleeding, alcoholic liver disease, chronic liver disease, and jaundice were also detectable. Similarly to the respiratory case, all of the hepatic phenotypes except necrosis of the liver also had a diagnosis rate of between 16% (necrotic liver disease) and 75% (oesophageal bleeding) of chronic liver disease. Renal failure achieved AUCs of 0.8 and 0.76, while end-stage renal disease achieved 0.83 and 0.83, consistent with previous results.^10^ And four manifestations of sepsis and septicaemia achieved AUCs of between 0.82 and 0.85 at Stanford and 0.77 and 0.81 at Cedars-Sinai, also replicating previous results.^12^

The model was successful in detecting a range of phenotypes related to abnormal white blood cell count. It detected acute lymphoid and myeloid leukaemia with AUCs of 0.86 and 0.83 at Stanford and 0.85 and 0.83 at Cedars-Sinai. It also performed well in detecting aplastic anaemia, pancytopenia, neutropenia, and bone marrow or stem cell transplants, all with AUCs between 0.82 and 0.83 at Stanford and 0.78 and 0.79 at Cedars-Sinai.

There were several metabolic conditions which the model successfully detected, for the most part unrelated to one-another. Protein-calorie malnutrition, disorders of the plasma protein metabolism, of the phosphorus metabolism, and of bilirubin excretion, fluid overload, and hyperosmolality were all detectable at Stanford with AUCs between 0.8 and 0.86 and at Cedars-Sinai with AUCs between 0.76 and 0.80. The phenotype for disorders of bilirubin excretion was mostly disjoint from jaundice, with only 45 of the 2369 patients with either condition having both.

Finally, an assortment of other conditions without apparent relations to each other were detectable. The models were successful in detecting several non-specific phenotypes, including mycoses, other and unspecified coagulation defects, delirium due to conditions classified elsewhere, and poisoning by agents primarily affecting blood constituents. It is likely that these were dominated by single specific diagnosis codes or sets of similar diagnosis codes related to more-specific changes in ECG. For example, examination of mycoses diagnosis codes showed that the majority of specific diagnoses were around aspergillus and other respiratory and systemic mycoses. Gangrene and disorders of menstruation were also easily detectable.

As a sensitivity analysis, we additionally consider which phenotypes would have been discovered with reversed criteria at Stanford and Cedars-Sinai, i.e. requiring an AUC of 0.75 at Stanford and 0.8 at Cedars-Sinai. The list of considered conditions is largely the same, and we discover only 5 different non-cardiac conditions: suicidal ideation, obstructive chronic bronchitis (associated with respiratory failure), cirrhosis of liver without mention of alcohol (associated with cirrohis of the liver), dementia (associated with old age), and pain and other symptoms associated with female genital organs (associated with female sex and disorders of menstruation). Supplementary material online, *Table S4* shows model performance for key phenotypes across three different window sizes for time between diagnosis and ECG, showing minimal difference in performance.

### PheWASNet outperforms a wide array of baselines for most diseases

While the PheWASNet models performed well across many different phenotypes, it is not clear to what extent this is a consequence of the power of artificial intelligence rather than the fact that there were previously unrecognized features in the ECG that allow for easy diagnosis. *Figure 1B–J* show a number of baseline experiments where we compare PheWASNet performance to a set of simpler detectors.

We first compared PheWASNet to linear and random forest models based on 555 numerical measurements extracted from the ECG, following our previous work.^22^ The linear model performed an average of.079 worse than PheWASNet, with PheWASNet doing at least 5% better in 84.1% of phenotypes and 10% better in 22.1% of phenotypes (*Figure 1B*). The random forest model performed on average.067 worse, with a 5% gap in 64.8% of phenotypes and a 10% gap in 15.0% (*Figure 1C*). For both baselines, no phenotype did more than 5% better. Cases where these baselines outperformed PheWASNet by a smaller margin included other instances of bundle branch and AV block, obesity, and amyloidosis, all of which have simple descriptions in the ECG (low voltage for the latter two).

We next compared the PheWASNet model to demographic baselines, since previous work has shown age, sex, and race are regressable from the ECG.^9,29^ We first evaluated age as a univariate predictor of each phenotype, allowing either older or younger age to correlate with disease (*Figure 1D*). PheWASNet performed 5% better in 85.9% of cases, 10% better in 62.2% of cases, and averaged a.144 improvement over age. The phenotypes where age beat PheWASNet by more than 5% included various manifestations of memory loss and dementia, fracture of the femur, and diverticulosis. Next we compared with a linear model based on age, sex, race/ethnicity, and BMI (*Figure 1E*). This model outperformed PheWASNet on 10.1% of phenotypes, including many around sex-specific disorders, disorders associated with old age, and specific cancers including bladder, skin, and breast. The only such cases where the PheWASNet achieved an AUC of above 0.75 were dementia, fracture of the femur, and lupus, and disorders of menstruation, the first two likely owing to strong signals of old age in the ECGs of patients with those conditions.

Next, to understand whether the PheWASNet model is predicting something specific about disease vs. evaluating overall health, we compared with the SEER score (*Figure 1F*). The SEER score is a previously published deep learning ECG-based risk score for long-term cardiovascular mortality which has been shown to be associated with a range of cardiovascular disease.^6^ PheWASNet performed 5% better in 83.4% of cases, 10% better in 41.0% of cases, and averaged 0.094 improvement over SEER. There were no cases where the PheWASNet model achieved an AUC of above 0.75 and the SEER model outperformed the PheWASNet model.

One way PheWASNet might accurately detect disease is by using a specific measurement from the ECG which is related to that disease (*Figure 1G–I*). We next compared with three specific measurements from the ECG, the heart rate, corrected QT interval (QTc), and T-wave area in aVR. The last was chosen as the measurement with the highest average AUC across all phenotypes which wasn’t directly related to heart rate or QTc. These measurements did not independently detect any diseases with AUC of 0.75, with the exception of QTc predicting left bundle branch block with an AUC of 0.77. Heart rate was most successful in detecting tachycardia (AUC 0.72), Sepsis (0.71), SIRS (0.71), respiratory insufficiency (0.71), and neutropenia (0.71). The QTc detected left bundle branch block (0.77), complete AV block (0.74), and reduced ejection fraction (0.72). The T-wave area in aVR detected cardiogenic shock (0.75), reduced ejection fraction (0.72), and shock (0.71). Other measurements with high AUCs in detecting certain conditions are shown in Supplementary material online, *Table S2*.

We compared the normal 12-lead PheWASNet to a model trained the same way using only lead I of the ECG (*Figure 1J*). The single-lead model was on average 0.55 worse at detecting phenotypes, with 54.5% having a >0.05 difference and only 4.4% having a greater than.1 difference. The most notable differences were prostate cancer, which the 12-lead model detected with AUC 0.74 vs. the single lead model’s 0.58, and disorders of menstruation, for which the difference was 0.84 vs. 0.71. Finally, we compared the trained PheWASNet to a model with the same architecture and randomly initialized weights (*Figure 1K*). The random model’s AUCs were on average 0.218 lower than the trained model. In all cases the trained model performed better, in 99.3% of cases by more than 0.05 and in 94.9% by more than 0.1.

### The phenotypes PheWASNet detects accurately can be categorized into explainable clusters

Among the 78 phenotypes which were most detectable, many were clearly highly related, so we set out to reduce the number of phenotypes to explore via grouping. First, we removed the 31 cardiovascular phenotypes, and then removed 10 phenotypes which were descendants in the phecode hierarchy of other detectable phenotypes, resulting in 37 phenotypes to cluster. We considered pairs of phenotypes which co-predicted each other, where the model prediction for each phenotype detected the other phenotype with an AUC at most 0.05 lower than its own model prediction. We selected four of the phenotypes most often occurring in these pairs as central phenotypes of four clusters, which we found as a group co-predicted all but two conditions (*Table 2*).

**Table 2:** The results of categorisation of non-cardiac high-performing phenotypes.

Respiratory failure, insufficiency, arrest		Neutropenia		Chronic liver disease and cirrhosis	
Overlapping disease	Overlap	Overlapping disease	Overlap	Overlapping disease	Overlap
Septicaemia	0.28	Mycoses	0.16	Disorders of plasma protein metabolism	0.14
Protein-calorie malnutrition	0.4	Lymphoid leukaemia, acute	0.37	Alcoholic liver damage	0.50
Hyperosmolality and/or hypernatremia	0.57	Myeloid leukaemia	0.33	Oesophageal bleeding (varices/hemorrhage)	0.75
Disorders of bilirubin excretion	0.31	Aplastic anaemia	0.30	Chronic liver disease and cirrhosis	1.00
Other and unspecified coagulation defects	0.36	Neutropenia	1.00	Ascites (non-malignant)	0.35
Delirium due to conditions classified elsewhere	0.47	Bone marrow or stem cell transplant	0.14	Liver replaced by transplant	0.48
Bacterial pneumonia	0.41			Jaundice (not of newborn)	0.13
Pneumonitis due to inhalation of food or vomitus	0.38				
Empyema and pneumothorax	0.39	**Renal failure**		**Unclustered**	
Pleurisy; pleural effusion	0.27	**Overlapping disease**	**Overlap**	**Lung transplant**	
Respiratory failure, insufficiency, arrest	1	Disorders of phosphorus metabolism	0.51	Disorders of menstruation	
Respiratory complications	0.59	Fluid overload	0.45		
Acute and subacute necrosis of liver	0.49	Pulmonary congestion and hypostasis	0.39		
Shock	0.41	Other pulmonary inflamation or oedema	0.34		
Poisoning by agents primarily affecting blood constituents	0.36	Renal failure	1.00		
Sepsis and SIRS	0.34	Gangrene	0.51		

Overlaps indicate the proportion among ECGs where each phenotype occurs where the central phenotype of the cluster also occurs.

One cluster, centred on neutropenia, contains the five phenotypes related to white blood cell abnormalities, including lymphoid and myeloid leukaemia, aplastic anaemia, and bone marrow transplant, as well as mycoses. Another, centred on chronic liver disease and cirrhosis, includes most manifestations of liver disease, excluding necrosis of the liver. The third is centred on renal failure and includes a range of related conditions including fluid overload, pulmonary congestion and inflammation, disorders of phosphorus metabolism, and gangrene. The final and largest cluster, centred on respiratory failure, insufficiency, and arrest, includes a wide range of respiratory conditions, as well as sepsis and septicaemia, coagulation defects, blood poisoning, and necrosis of the liver. Two phenotypes remained unclustered, lung transplant and disorders of menstruation. With these clusters we have the 78 disease phenotypes to six ECG phenotypes to investigate, the four clusters and two unclustered diseases. Co-detection between cardiac and non-cardiac diseases was limited, with the exception of cardiac arrest and ventricular fibrillation, which were co-detectable with 12 of the 37 highly-detectable diseases. Fluid overload was also co-detected with pulmonary heart disease, and pulmonary congestion with congestive heart failure (CHF).

The model’s performance in detecting disorders of menstruation is likely due to the phenotype occurring almost exclusively in younger women. Age on its own achieves an AUC of 0.82 in detecting disorders of menstruation, and patients with the disorder have an average age of 41 (standard deviation 13) vs. patients without it with a mean age of 62 (18). Adding in demographics (including sex) achieves an AUC of 0.92. Patients with right atrial enlargement had 7.7 times higher odds of having a diagnosis code for lung transplant, and 6.5 times higher odds of being in the top 5% of risk according to the PheWASNet model. No other condition was explained well by demographics or any other baseline, or had an odds ratio greater than five given any ECG abnormality.

The linear model baseline of 555 measurements was successful in detecting neutropenia (0.79 vs. 0.83 PheWASNet), lung transplant (0.84 vs. 0.89), and disorders of menstruation (0.78 vs. 0.84), suggesting that it might be possible to design simpler linear models for detecting these phenotypes. Using lasso and manual removal of correlated and non-intuitive measurements we developed simple linear models with seven or fewer inputs to detect each condition (*Table 3*). Neutropenia was detectable with an AUC of 0.76 from just four measurements, the heart rate and QRS duration, Q wave duration, and P + P′ durations in lead aVF. The same small linear model detected lymphoid and myeloid leukaemia, aplastic anaemia, and bone marrow or stem cell transplants with AUCs between 0.71 and 0.75. Respiratory failure was detectable with an AUC of 0.75 using five measurements, the heart rate, QTc dispersion, T-wave amplitude in aVR, peak-to-peak aVR QRS magnitude, and P + P′ durations in V4. That model could detect each phenotype in its group with an AUC of between 0.70 and 0.80, with shock (0.80), poisoning by agents primarily affecting blood constituents (0.78), and sepsis (0.76) being the highest. Lung transplant was detectable with an AUC of 0.77 with just 6 measurements, while disordered menstruation was detectable with an AUC of 0.78 and 7 measurements. Ascites was detectable with an AUC of 0.75 using just the peak-to-peak V5 QRS magnitude and heart rate, but that model did not generalize well to chronic liver disease and cirrhosis, where it achieved an AUC of 0.66.

**Table 3:** Simple linear models for four conditions, with performance in the Stanford cohort

Task	AUC (PheWASNet)	AUC (linear model)	AUC (simple linear model)	Measurement	Weight
Neutropenia	0.83	0.79	0.76	Number of QRS Complexes	0.55
				QRS Duration in aVF	−0.36
				P + P′ Duration in aVF	−0.24
				Q Amplitude in aVF	0.17
				Intercept	−4.88
Respiratory Failure	0.86	0.78	0.75	Number of QRS Complexes	0.53
				QTc dispersion	0.25
				T Amplitude in aVR	0.24
				QRS total peak to peak amplitude in aVR	−0.21
				P + P′ Duration in V4	−0.20
				Intercept	−3.52
Lung transplant	0.89	0.84	0.77	Mean QTc	−0.63
				PR interval in aVR	−0.51
				PR segment in V2	−0.38
				S Amplitude in aVL	−0.27
				P amplitude in II	0.26
				Number of QRS Complexes	0.05
				Intercept	−6.30
Disorders of menstruation and other abnormal bleeding from female genital tract	0.84	0.78	0.78	QRS total peak to peak amplitude in V4	−0.72
				PR interval in aVR	−0.40
				T Amplitude in I	0.39
				Mean QRS duration	−0.23
				R amplitude in II	0.20
				P + P′ Duration in V6	−0.20
				S amplitude in V1	−0.15
				Intercept	−6.75
Ascites	0.85	0.77	0.75	QRS total peak to peak amplitude in V5	−0.64
				Number of QRS Complexes	0.40
				Intercept	−5.61

## Discussion

In this work we have presented a set of disease phenotypes detectable from the ECG using PheWASNet, a deep learning model, and attempted to understand what ECG features likely lead to their detectability. We confirmed previous results on chronic kidney disease, liver disease, sepsis, and a range of cardiovascular disease, while also discovering new links to respiratory failure, white blood cell-related disorders, lung transplant, and a range of other disease. We developed simple models to understand how the ECG might detect some of these phenotypes, and compared with a battery of baselines to understand how complicated each phenotype is.

We identified 37 non-cardiovascular diseases which our model could successfully predict. Some were previously known to have effects on the ECG: sepsis has previously been associated with lower QRS amplitude and higher QRS duration,^30^ lung transplant with atrial arrhythmias,^31^ and respiratory distress and failure with a number of ECG changes.^32^ Other conditions were more surprising. Neutropenia in particular is interesting in that, to our knowledge there has been little to no research into the effect of white blood cell disorders on the ECG.

Additionally, these 37 diseases fit into six groups of co-predictable phenotypes. One, disorders of menstruation, could be explained entirely by demographic information partially inferable from the ECG. The existence of such phenotypes is perhaps unsurprising, but serves as a reminder for why strong and varied baselines are necessary in the deep learning setting. Lung transplant, neutropenia, and sepsis were detectable by simple linear model, offering a window into which features of the ECG are useful in identifying these disease states. Lung transplantation was additionally strongly associated with right atrial enlargement. For chronic kidney disease and cirrhosis of the liver, two diseases previously known to be detectable from the ECG,^10,11^ we were not able to identify features in the waveform which might be useful for detection. In the case of cirrhosis, ascites detection can be mostly explained based on heart rate and QRS magnitude in V5, but that result does not generalize to cirrhosis overall. The use of co-predictability demonstrates both a discovery and a limitation of the work. On the one hand, we significantly reduced the number of diseases to understand, and found both obvious and surprising connections between conditions. On the other hand, the fact that these conditions can be predicted using similar features shows that those features might not be useful in distinguishing between the conditions.

This work is only possible due to the double-edged sword of high-throughput phenotype generation from the electronic health record. On the one hand this technique allows for the automation of label generation, allowing thousands of labels to be generated in the same time it would take to generate one. This allows for a view across organ systems and the entire range of disease. On the other, these labels are noisier and lower-quality than those curated for a specific task. Label curation can have a significant impact on results, and ICD-9 and 10 based labels in particular can be noisy.^33^ For instance, our PheWASNet model detected heart failure with low ejection fraction, labelled based on ICD-9 and 10 code, with an AUC of 0.87, vs. an AUC of 0.94 when defining the phenotype using measurements taken directly from matched echocardiogram notes.^22^ Often, no gold standard diagnosis tool is available for a specific disease, and diagnosis codes are the only available label for a given disease, and at scale the task of collecting a wide variety of gold standard tests for thousands of diseases is impractical. When a gold standard test is available, there isn’t always a clear machine-interpretable record of that gold standard test, as was the case for HFrEF where echocardiogram notes had to be carefully parsed to extract labels. When continuous values are available, regressing them rather than performing a classification can also improve performance.^3^ A similar problem applies to exclusion criteria: if one wants to develop a screening or risk tool for a specific disease, the cohorts that tool is tested in should carefully reflect the screening population, but in this high-throughput setting, all phenotypes have the same inclusion criteria. In that setting, both the label providence and the inclusion criteria are radically different, Similarly bundle branch and AV blocks based on diagnosis code were detected with AUCs between 0.84 and 0.9, much lower than what one would expect if the labels were generated by direct ECG overread.^1^ This gap is easy to observe in cardiovascular conditions, but harder in novel non-cardiac conditions where there is no clear upper baseline. Thus, the performance throughout this work might be biased downwards. It is our hope that future work takes the insights of this work and explores specific phenotypes in greater detail, with focus on high label quality and cohort design.

This project differs from previous PheWAS studies in two notable ways. First, rather than discovering direct associations between some measurement or simple trait and a list of labels, we instead trained a deep learning model on a complex data modality and analyzed which labels were detectable. This makes replicating results at a second site all the more important due to the risk of overfitting. Second, we found a very high rate of significant associations, with 1110 of the 1243 phenotypes having some association with the ECG. This number is high, but perhaps not surprising, as age, gender, race, and a wide range of other comorbidities all have known effects on the ECG. We also employed a novel multi-site model evaluation scheme, training independent models at each site rather than training at a primary site and evaluating at others. We chose this setup to test whether trends can be independently replicated across training and evaluation data from multiple sites, rather than assessing a single model’s ability to generalize across medical systems; in other words, the end result of our evaluation is the set of replicably detectable PheCodes, not a single model. An advantage of this approach is that results at secondary sites aren’t biased by the usual loss in performance which is observed during cross-site evaluation.^22^ Models for clinical screening for individual phenotypes could be trained in the future and should be evaluated in a more traditional fashion.

Our work has limitations. As high-throughput phenotyping presents one challenge, high-throughput modelling presents another: whereas when training a model for a single task, optimisation decisions are made specifically for that task, but when training a model for a thousand tasks, decisions can’t be made on a task by task basis. Interpretability remains a challenge, and although we make some strides in understanding the phenotypes our models detect well, there still is a large gap between our work and a full comprehension of model mechanisms. While we present some positive results, our failure to detect other phenotypes does not necessarily prove that those phenotypes aren’t detectable at all, just that our modelling in our datasets cannot detect them. And while we present results from two separate large medical centres, our results won’t necessarily generalize to other sites and settings, especially those with significant demographics and comorbidities.^34^

We have presented a range of non-cardiac disease phenotypes detectable from the ECG and made efforts to understand what features of the ECG allow this. Our hope is that these findings serve as a starting point for further investigation into these specific phenotypes, how they influence the ECG and the heart, and whether there are opportunities to screen for them from the ECG.

## Supplementary Material

ztaf047_Supplementary_Data

## Data Availability

Patient data and model weights beyond what is shared in this article cannot be shared due to constraints around patient privacy.
