# Supplementary material for: A deep learning phenome wide association study of the electrocardiogram
Source: Eur Heart J Digit Health. 2025 May 8;6(4):595–607. doi: 10.1093/ehjdh/ztaf047 (PMC12282379; doi:10.1093/ehjdh/ztaf047)
Supplement: ztaf047_Supplementary_Data [file ztaf047_supplementary_data.zip › Supplemental Tables and Figures.docx]

# Supplemental Tables and Figures

## Supplemental Table 1

ECG counts and demographics at each center.

|  | Stanford | Cedars-Sinai |
| --- | --- | --- |
| Train ECGs (patients) | 382729 (127193) | 538091 (213298) |
| Valid ECGs (patients) | 95654 (32005) | 134455 (88143) |
| Test ECGs (patients) | 476434 (159118) | 674245 (241417) |
| Total ECGs (patients) | 954817 (318316) | 1346791 (542858) |
|  |  |  |
| Non-Hispanic White | 804570 (56.7%) | 777235 (57.0%) |
| Asian | 145635 (10.3%) | 81780 (6.0%) |
| Black or African American | 77047 (5.4%) | 224178 (16.4%) |
| Hispanic White | 30655 (2.2%) | 138465 (10.2%) |
| Other race | 20904 (1.5%) | 109663 (8.0%) |
| Unknown race | 340031 (24.0%) | 32722 (2.4%) |
| Male | 743847 (52.4%) | 707133 (51.8%) |
| Age | 61 (17) | 64 (18) |

##

## Supplemental Table 2

The highest AUCs of single measurements in detecting any phenotypes in the Stanford dataset.

| **Phenotype** | **measurement** | **AUC** |
| --- | --- | --- |
| Right bundle branch block | transqrstermangle | 0.91 |
| Left bundle branch block | V4_qrsdur | 0.87 |
| Bundle branch block | I_qrsdur | 0.86 |
| Atrioventricular block, complete | I_qrsdur | 0.79 |
| Amyloidosis | II_ramp | 0.77 |
| Cardiogenic shock | aVR_qrsppk | 0.76 |
| Tachycardia NOS | numberofcomplexes | 0.75 |
| Disease of tricuspid valve | aVR_parea | 0.75 |
| Systemic inflammatory response syndrome (SIRS) | numberofcomplexes | 0.74 |
| Sepsis and SIRS | numberofcomplexes | 0.74 |
| Heart transplant/surgery | numberofcomplexes | 0.74 |
| Sepsis | numberofcomplexes | 0.74 |
| Poisoning by agents primarily affecting blood constituents | aVR_qrsppk | 0.74 |
| Lung transplant | II_qtint | 0.74 |
| Heart failure with reduced EF [Systolic or combined heart failure] | I_tptparea | 0.74 |
| Neutropenia | numberofcomplexes | 0.73 |
| Respiratory insufficiency | numberofcomplexes | 0.73 |
| Other pulmonary inflamation or edema | I_tarea | 0.73 |
| Atrioventricular [AV] block | V3_qrsdur | 0.73 |
| severe protein-calorie malnutrition | numberofcomplexes | 0.73 |
| Septicemia | numberofcomplexes | 0.73 |
| Acute and subacute necrosis of liver | numberofcomplexes | 0.72 |
| Shock | numberofcomplexes | 0.72 |
| Ascites (non malignant) | V5_qrsppk | 0.72 |
| Decreased white blood cell count | numberofcomplexes | 0.72 |
| Lymphoid leukemia, acute | highprint | 0.72 |
| Debility unspecified | numberofcomplexes | 0.72 |
| Congestive heart failure; nonhypertensive | I_tptparea | 0.72 |
| Endocarditis | meanqtc | 0.72 |
| Congestive heart failure (CHF) NOS | I_tptparea | 0.72 |

## Supplemental Table 3

Supplemental Table 3 is large and is attached as an excel spreadsheet, [and is available here for download from DropBox](https://www.dropbox.com/scl/fi/uxizznya7dklhpmzn7t9n/STable3.xlsx?rlkey=7sro1p3u6shqb31idtrf9npsp&st=v6gigz1w&dl=0). Performance of Stanford, Cedars-Sinai, and Stanford baseline models on all phenotypes with more than 500 examples at each site, along with counts. AUC: area under the ROC curve. AUPRC: area under the precision-recall curve. TPR: true positive rate. TNR: true negative rate. FPR: false positive rate. FNR: false negative rate.

## Supplemental Table 4

AUCs of the PheWASNet model trained on labels constructed with a 30 day window and evaluated on labels constructed with 7, 30, and 90 day windows for diagnosis codes around ECGs.

| PheCode | AUC (7-day window) | AUC (30-day window) | AUC (90-day window) |
| --- | --- | --- | --- |
| 288.11 | **0.836** | 0.828 | 0.812 |
| 429.1 | 0.916 | 0.926 | **0.927** |
| 509 | 0.85 | **0.857** | 0.852 |
| 510.2 | 0.872 | **0.889** | 0.886 |
| 571 | **0.802** | 0.801 | 0.79 |
| 585 | 0.79 | **0.804** | 0.801 |
| 626 | 0.839 | **0.843** | 0.842 |
| 994.2 | 0.847 | **0.848** | 0.837 |

##

## Supplemental Figure 1

An example of co-detection. The PheWASNet model trained to detect respiratory failure does so with an AUC of 0.86. The PheWASNet model trained to detect sepsis does so with an AUC of 0.84. The respiratory failure model detects sepsis with an AUC of 0.84, and the sepsis model also detects respiratory failure with an AUC of .0.84. Because both “cross”-detections are within 0.05 of the normal detection, we refer to the two as co-detecting each other. Note that sepsis and respiratory failure have an overlap of 17%, so are related but certainly not identical populations.


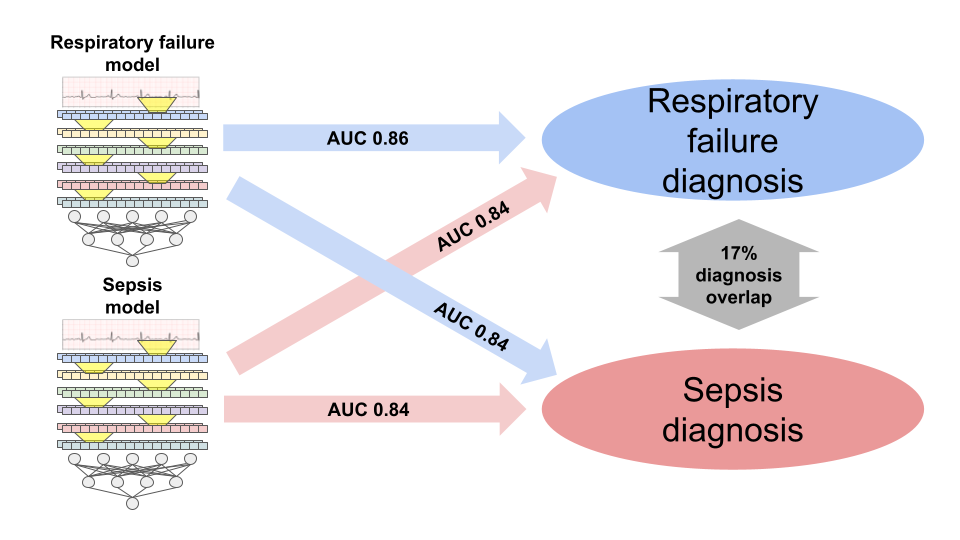


## Supplemental Figure 2

Proportion of ECGs of each phenotype on the vertical axis which also has the phenotype on the horizontal axis.


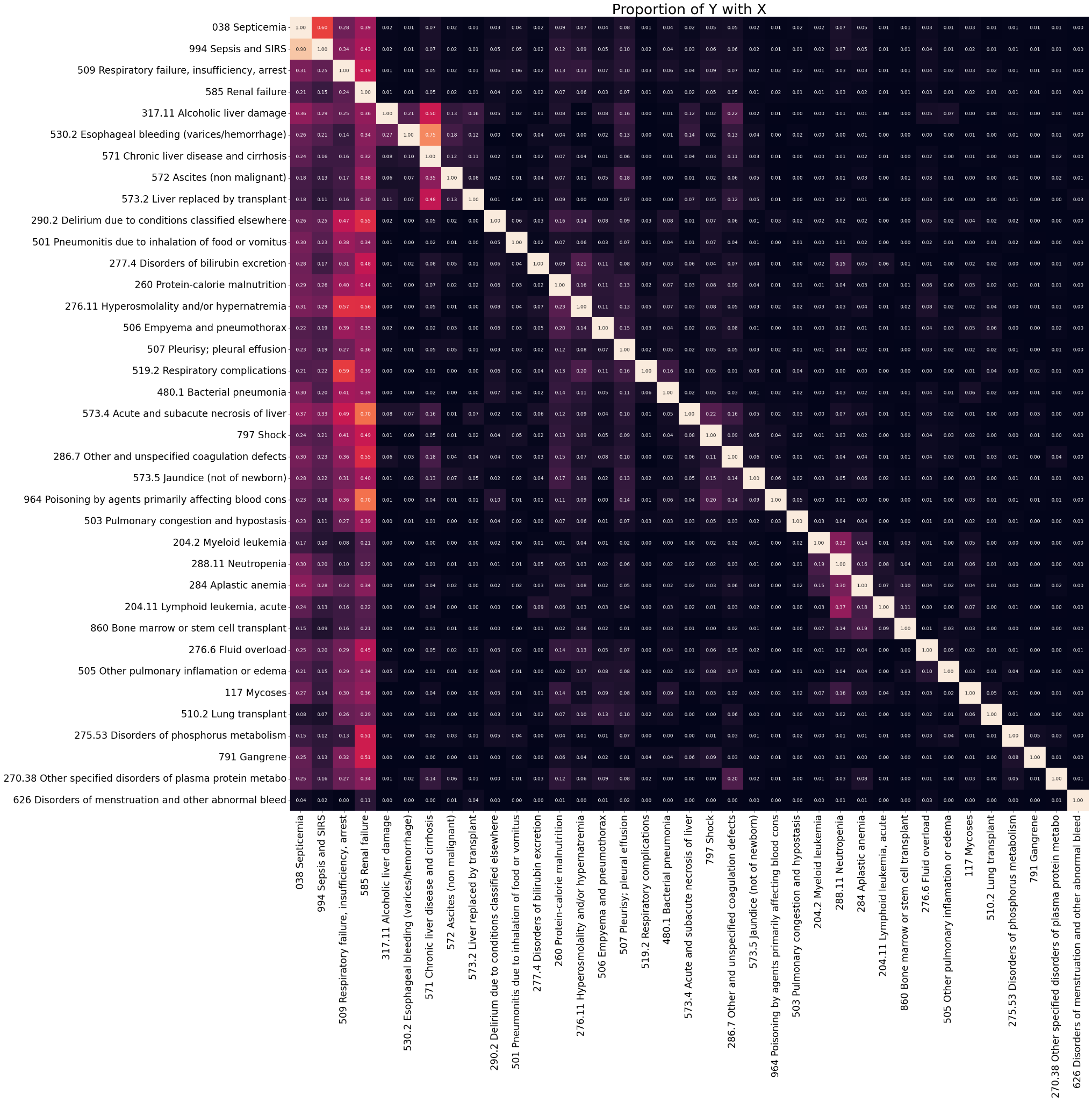


## Supplemental Figure 3

PheWASNet model architecture. FC: fully connected layer. MBConv: Mobile Inverted Bottleneck Convolution. ReLU: Rectified Linear Unit. ReLU6: Rectified Linear Unit 6.


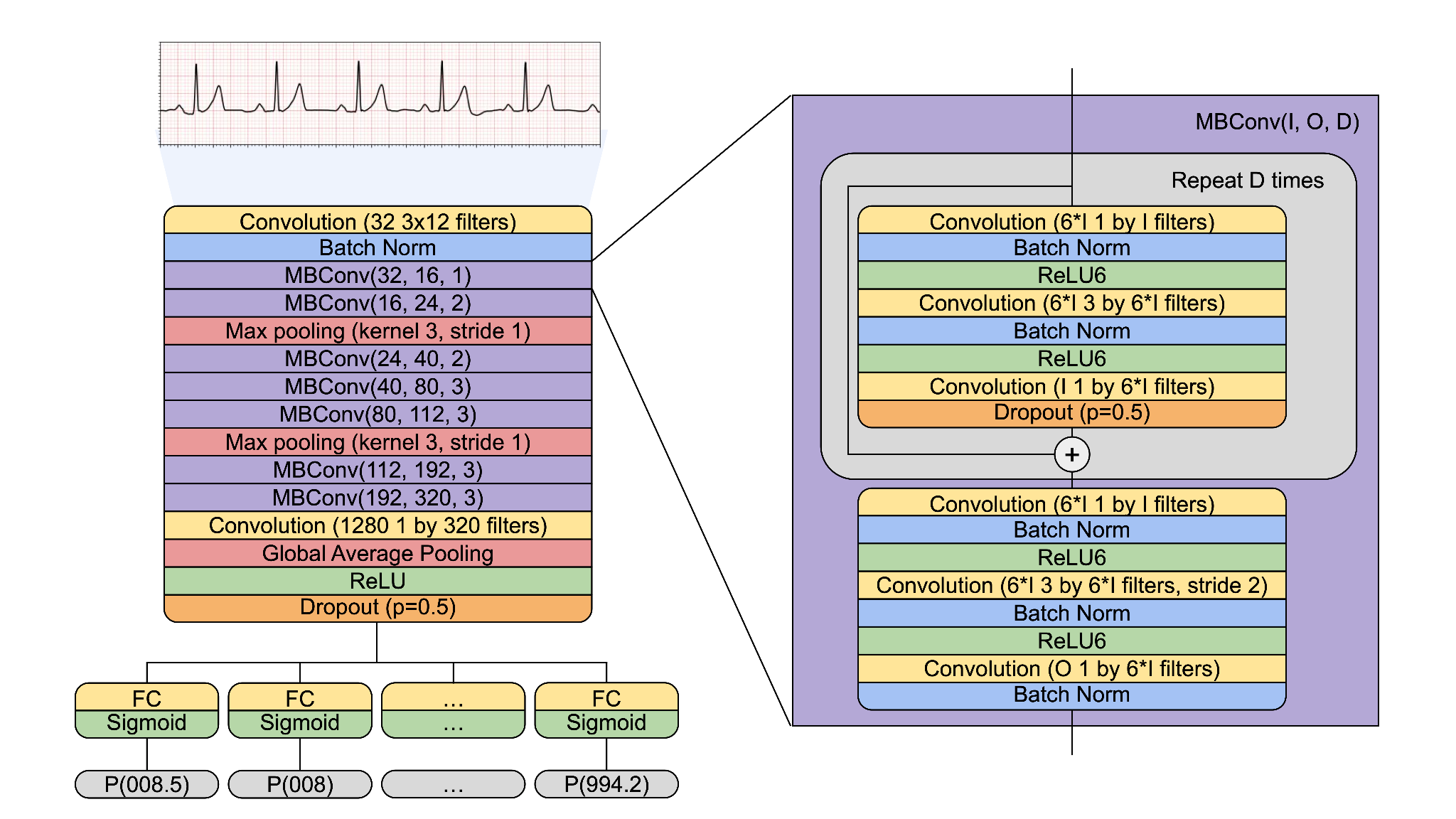


## Supplemental Figure 4

AUROC curves for four selected conditions, along with sensitivities achieved at three key specificity thresholds. AUC: area under the ROC curve.


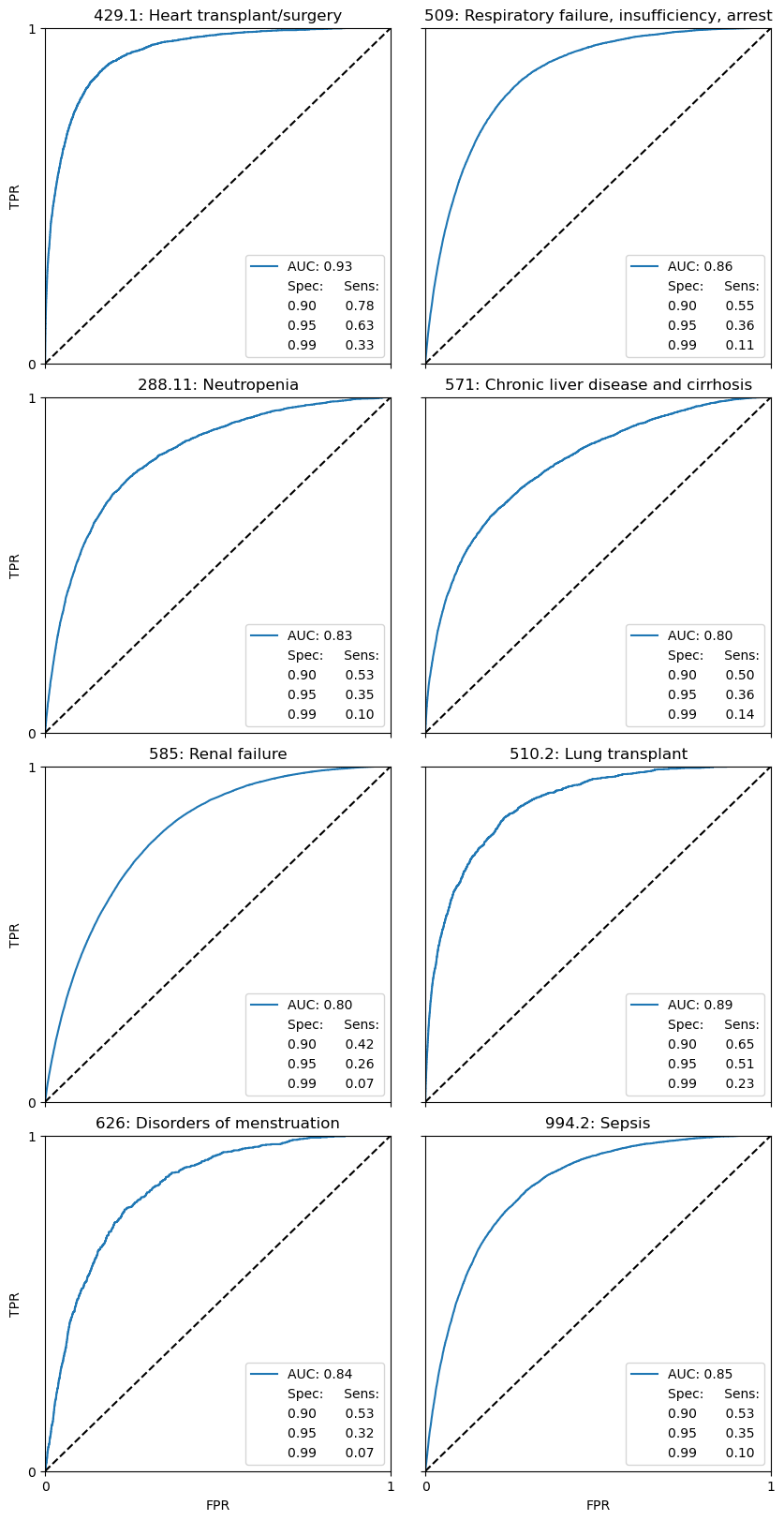


## 
